# Nickel-Loaded 3D-Printed Electrode for In Situ Electrochemical Conversion to a Prussian Blue Analogue: Synthetic Parameter Optimization for Pseudocapacitor Applications

**DOI:** 10.1021/acsmaterialsau.5c00025

**Published:** 2025-05-28

**Authors:** Pedro H. S. Borges, Natália M. Caldas, Lucas V. de Faria, Rafael M. Dornellas, Edson Nossol

**Affiliations:** † Institute of Chemistry, 28122 Universidade Federal de Uberlândia, Uberlândia 38408-902, Brazil; ‡ Department of Analytical Chemistry, Institute of Chemistry, 28110 Universidade Federal Fluminense, Niterói 24020-141, Brazil; § Department of Analytical Chemistry, Institute of Chemistry, Federal University of Rio de Janeiro, Rio de Janeiro 21941-909, Brazil

**Keywords:** pseudocapacitor, 3D printing, energy storage, nickel hexacyanoferrate, Prussian blue analogue

## Abstract

Developing cost-effective and scalable energy storage devices is critical for advancing sustainable technologies. This study presents the fabrication of a novel 3D-printed PLA/Gr/NiHCF electrode, leveraging the benefits of additive manufacturing and a systematic factorial design of experiments (DOE) approach. The motivation stems from the need for simplified production methods that deliver high-performance materials while reducing waste and energy consumption. The electrode was synthesized through a two-step process involving 3D printing of a PLA/graphite/nickel acetate (PLA/Gr/Ni) composite followed by electrochemical conversion of nickel hexacyanoferrate (NiHCF) particles. The factorial DOE methodology optimized the composition of the PLA/Gr matrix and the electrochemical deposition conditions, ensuring a robust process with reproducible outcomes. The structural and electrochemical properties of the materials were evaluated using FTIR, Raman, SEM, EDS, CV, and EIS. The PLA/Gr/NiHCF electrode exhibited outstanding electrochemical performance, with a specific capacitance (C_s_) of 37.33 mF cm^–2^ at 0.1 mA cm^–2^ in a three-electrode system, significantly outperforming the control PLA/Gr electrode (0.58 mF cm^–2^). In a two-electrode symmetrical configuration, the system delivered a C_s_ of 40.4 mF cm^–2^ at 0.1 mA cm^–2^, with excellent retention (95% over 100 cycles) and reversible Coulombic efficiency (98.3%). The electrode’s pseudocapacitive behavior, driven by the surface-confined redox activity of NiHCF, was confirmed through CV and EIS analyses. The results highlight the practicality of 3D printing combined with simple electrochemical modification for producing efficient supercapacitor electrodes. This study underscores the importance of factorial DOE in optimizing material properties and establishes the PLA/Gr/NiHCF electrode as a promising candidate for scalable, sustainable energy storage applications.

## Graphical abstract

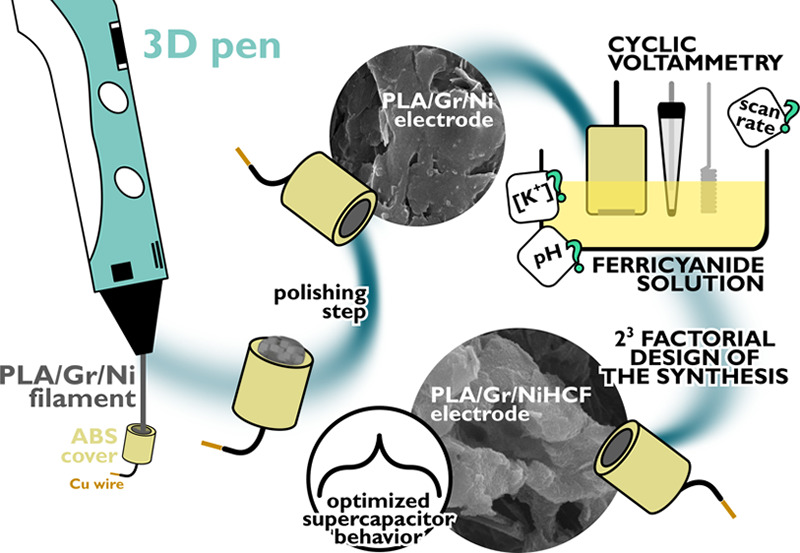

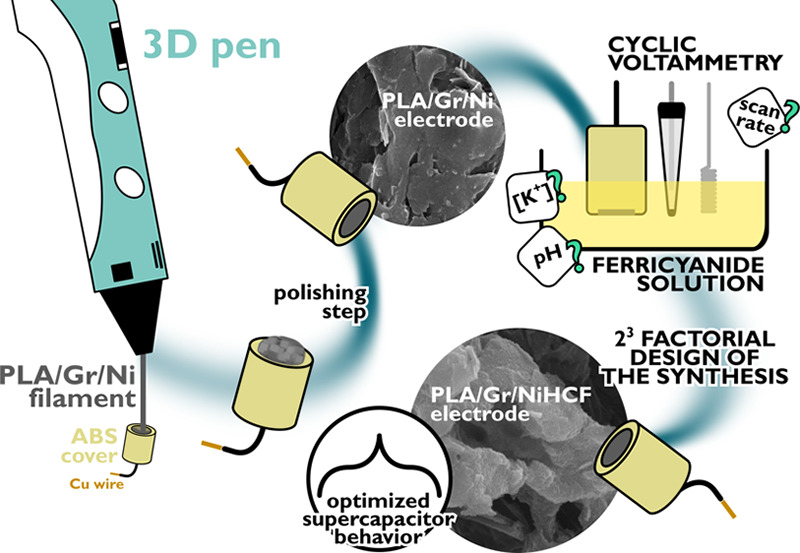

## Introduction

The heavy demand for novel clean energy production methods arises alongside the need for efficient storage systems. These devices must adhere to green parameters that are aligned with sustainable technological development.^1,2^ Supercapacitor systems have been extensively explored as promising environmentally friendly alternatives due to their ability to achieve high power density, charge/discharge reversibility, and durable cyclability in aqueous media. The long lifespan of these devices is strongly linked to surface-confined redox activity, which avoids phase transformation during the ionic intercalation process. This phenomenon decomposes electrode materials in purely faradaic systems, such as batteries.^3–5^ However, this rapid process reduces the overall energy density of the component. As a result, supercapacitors are highly recommended as complementary components for fuel cells and rechargeable batteries.^6^ Since the energy storage capacity of a material is intrinsically associated with its properties, strategic design and synthesis of new materials are crucial for improving efficiency while minimizing environmental impact.

Prussian blue (PB) and its analogues (PBAs) have demonstrated notable performance in supercapacitor systems due to their zeolite-like crystalline structure and active redox sites. PB comprises Fe^II^ and Fe^III^ sites octahedrally bridged by cyano ligands, while PBAs retain the same structure but incorporate other transition metals in place of iron. PBA can exist in forms called “soluble” and “insoluble”, although both are highly insoluble in aqueous media, the name refers to the former being slightly more dispersible. The former is an interstitial cation (usually an alkali metal) in the structure, whereas the latter is defect-rich due to [Fe­(CN)_6_]^3‑/4–^ occupied by coordinated water, leading to different chemical and electrochemical characteristics. In PBAs, one or both metal sites are typically electrochemically active, undergoing redox reactions within the structure without framework decomposition. Additionally, their fine atomic arrangement includes interstitial gaps forming 3D channels that facilitate ion and molecule transport.^5,7^ These properties make PBAs versatile materials for various applications, including sensing,^8,9^ catalysis,^10,11^ electrochromic devices,^12^ and energy storage systems, like batteries and supercapacitors.^5,13,14^ By tuning their composition and structure through controlled synthesis, it is possible to optimize their performance for specific applications.

Various methods can be employed to synthesize PB and PBAs, including coprecipitation,^15,16^ microemulsion,^17^ hydrothermal,^18,19^ and electrodeposition techniques.^20,21^ Among these, electrodeposition stands out for its simplicity and the ability to finely tune product properties. This method enables precise control over PBA particle morphology, size, and distribution while allowing structural and compositional adjustments. Material properties can be tailored by varying synthesis conditions such as precursor solution composition (concentration of active species, supporting electrolyte, and pH) and electrochemical parameters (potential, current, charge, and time).^21–23^ This high level of controllability makes electrodeposition particularly suitable for optimizing materials for specific operational requirements. Additionally, the factorial design of the experiments (DOE) serves as a powerful tool for synthesizing optimized materials by determining fabrication conditions and analyzing variable interactions.^24,25^

The nickel analogue of PB, known as nickel hexacyanoferrate (NiHCF), has shown promising performance in aqueous supercapacitor systems due to its stable and reversible redox activity in neutral electrolytes.^20,26^ These properties are closely tied to the morphological and structural features of the material, which can be effectively tuned through synthesis methods, particularly electrochemical deposition. For instance, in 2003, Zamponi and collaborators^22^ demonstrated how electrochemical deposition conditions significantly influence NiHCF formation. Their study revealed that higher static potentials favor the formation of “insoluble” NiHCF, while dynamic sweeping at lower potentials yields different results. Furthermore, the time of static potential application and K^+^ electrolyte concentration were shown to affect structure formation. Similarly, Ma and colleagues^20^ fabricated NiHCF nanocubes on carbon fibers using potential pulses and demonstrated how pulse parameters control the structural and morphological features, including particle size, distribution, and uniformity.

Incorporation of strategic materials in fused deposition modeling, (FDM) 3D-printed-based electrodes is highly attractive due to the low cost of production and effortless operation. Commercial filaments of PLA/graphene had their energy storage performance evaluated by Pumera’s research group. They verified that the presence of remaining metal species from graphene synthesis was responsible for the superior performance of the electrode, as they were removed, the material showed expressive capacitance loss.^27^ In another work, they produced a filament composed of active carbon, multiwalled carbon nanotubes, MoS_2_, PLA, and polyethylene glycol with good printability, reaching 391 mF cm^–2^ (at 1.8 mA cm^–2^ current density).^28^ Both examples demanded post-treatments—a thermal procedure for the former, and a chemical treatment for the latter—to improve exposure of the active materials to the electrolyte, removing insulanting PLA content. This describes the main issue associated with FDM fabrication of electrodes, along with the difficult printability of inorganic species-incorporated filaments.^29,30^

Here, we present an in situ derivatization of a 3D-printed polylactic/graphite/nickel (PLA/Gr/Ni) electrode to prepare PLA/Gr/NiHCF, guided by a multivariate 2^3^ factorial DOE to evaluate the effects of variables and their interactions on charge storage efficiency. The material was synthesized via cyclic voltammetry (CV), examining the scan rate, K^+^ concentration in the electrolyte, and pH value. This approach enabled the identification of optimal conditions for supercapacitor applications. The optimized material was characterized alongside PLA/Gr and PLA/Gr/Ni electrodes, and its electrochemical performance was evaluated in comparison to the PLA/Gr control material. The results demonstrated pseudocapacitive behavior with reliable performance in both three-electrode and symmetrical two-electrode systems, considering the simplicity and efficiency of the production method.

## Experimental Section

### Reagents and Solutions

Deionized water was used to prepare the solutions employed in this work. Potassium chloride (≥99%) and hydroxide (≥85%) were purchased from Dinâmica (Brazil), while potassium nitrate (≥99%) was acquired from Quimex (Brazil). Hydrochloric acid (37%) and potassium ferricyanide (≥99%) were obtained from Êxodo Científica (Brazil). Acetone (99.5%) and chloroform (99.8%) were purchased from Synth (Brazil), and graphite (Gr) powder (98%, particle diameter <20 μm) was obtained from Sigma-Aldrich (USA). Nickel acetate tetrahydrate (99%) was purchased from Vetec (Brazil). The polymers acrylonitrile butadiene styrene (ABS) filament and polylactic acid (PLA) pellets were acquired from GTMax (Brazil) and 3DLAB (Brazil), respectively.

### Instrumentation

Thermogravimetric analysis (TGA) was conducted using a DTG-60H thermobalance (Shimadzu, Japan) under a 10 °C min^–1^ heating rate and air atmosphere. Scanning electron microscopy (SEM) images of the materials were acquired in a Vega3 microscope (Tescan, Czech Republic) under a voltage of 20 kV, and the elemental prediction was performed by energy-dispersive X-ray spectroscopy (EDS) using an INCA X-Act X-ray detector (Oxford Instruments, UK). For those analyses, the materials were coated with a 10 nm gold film to avoid PLA decomposition by the electron beam. Fourier-transform infrared spectra (FTIR) were obtained by a Frontier MIR/FIR spectrometer (PerkinElmer, USA) assisted by an attenuated total reflectance accessory (Pike Technologies, USA). Furthermore, Raman spectra were acquired under a 5% potency incidence of an Ar-ion laser (532 nm) operated by a LabRAM HR Evolution microscope (Horiba, Japan).

### Electrochemical Measurements

The electrochemical preparation of the PLA/Gr/NiHCF electrode and all electrochemical evaluations were conducted using a Squidstat Solo potentiostat/galvanostat (Admiral Instruments, USA). The only exception was for the electrochemical impedance spectroscopy (EIS), which was performed in a PGSTAT204 potentiostat/galvanostat (Metrohm, Switzerland) equipped with an EIS module. The reference electrode utilized was an Ag_(s)_/AgCl_(s)_/Cl^–^_(sat.)_ fabricated in the laboratory^31^ and the auxiliary electrode employed was a platinum wire. The working electrodes, with a geometric area of 0.196 cm^2^, included PLA/Gr, PLA/Gr/Ni, and PLA/Gr/NiHCF electrodes fabricated in this work. Their preparations are discussed in the following sections. Photographic images of the three- and two-electrode (symmetrical) setups are depicted in Figure S1a,b, respectively.

### Preparation of the PLA/Gr/Ni Electrode

All composite materials were prepared to a total mass of 30 g. The mixture between the filament components was initiated by the dissolution of PLA in 180 mL of a 3:1 (v:v) acetone:chloroform solvent mix for 1 h under a 70 °C reflux and vigorous stirring (5000 rpm). After that, a defined amount of the conductive components, Gr, and nickel acetate, were incorporated into the PLA organic solution and left to stir for another hour. Specifically, the control material (PLA/Gr, 70:30 *wt %*) was prepared using 9 g of Gr and 21 g of PLA. In contrast, the nickel-loaded composite (PLA/Gr/Ni, 57.5:30:12.5 wt %) was formulated with 17.25 g of PLA, 9 g of Gr, and 3.75 g of Ni. The solid mixture was dried over parchment paper at room temperature for a full day. The material was fragmented and added in a filament extruder (Filmaq 3D, Curitiba, Brazil) operated at full motor speed (30 rpm) at 200 °C. As a result, this procedure produced approximately 2 m of cylindrical filaments with a diameter of 1.75 mm, which were safely stored at room temperature inside plastic bags.

The prepared filament materials were loaded into 3D-printed ABS cylindrical templates using a 3D pen (Sanmersen, Shenzhen, China) operated at 190 °C. The electrical insulator ABS mold was designed using Solidworks 3DCAD software and sliced using Creality software. The template consisted of an inner hole of 0.5 mm diameter and an outer one of 0.75 mm diameter. The cylinder was printed by a Creality K1 FDM printer (Shenzhen, China) with 4.0 cm in length using the printing parameters exhibited in Table S1. The 0.5 mm diameter hole was filled with filament materials in the presence of a copper wire to ensure electrical contact between the conducting material and the potentiostat/galvanostat equipment. Finally, the proposed electrodes were polished with sandpaper of 600 and 1200 mesh, resulting in a 0.196 cm^2^ geometric surface area.

### Conversion of the PLA/Gr/NiHCF Electrode

The conversion of the Ni precursor to obtain its PBA in the PLA/Gr matrix was conducted by CV between 0 and 1 V in a 1.0 mmol L^–1^ potassium ferricyanide solution in KCl. The concentration of the supporting electrolyte ([K^+^]), the pH of the solution (pH), and the scan rate (υ) effects on the conversion of the matrix-trapped Ni^2+^ to NiHCF were examined by a 2^3^ factorial DOE with a triplicate in the center point (0) to obtain the best conditions for the proposed application. The low (−1) and high (+1) levels of the factors employed can be seen in Table 1. The number of cycles performed was determined by the υ to keep the time of conversion constant (4000 s). Therefore, 20, 110, and 200 cycles were applied for the levels of the υ of 10, 55, and 100 mV s^–1^, respectively. The full factorial DOE resulted in 11 experiments that are shown in Table S2 along with the codes of the factors: υ – scan rate; [K^+^] – concentration of the supporting electrolyte; pH – pH value of the ferricyanide/electrolyte solution, which was adjusted using concentrated HCl and KOH solutions. The result performance indicator chosen was the specific capacitance (C_s_) obtained at each experiment by the area of CV at 10 mV s^–1^ from 0 to 1 V in a 1.0 mol L^–1^ KCl solution. The C_s_ was calculated according to eq S1, where *j* is the current density (A cm^–2^), Δ*V* is the potential window (V), and υ is the scan rate (V s^–1^).^32^ The results, followed by the CV profiles obtained for each experiment, are depicted in Figure S2.

**Table 1:** Factors and Levels Employed in the 2^3^ DOE for PLA/Gr/NiHCF Conversion Optimization

	Level		
Factor	(−1)	(0)	(+1)
Scan rate (υ)/mV s^–1^	10	55	100
[K^+^]/mol L^–1^	0.1	0.55	1.0
pH_HCF_	1.0	4.0	7.0

## Results and Discussion

### Conversion of the PLA/Gr/NiHCF Electrode

The CV profiles shown in Figure S2 yielded C_s_ values, which are summarized in Figure 1a using a geometric representation of the effects from the 2^3^ factorial DOE. Experiment 2, conducted at a υ of 100 mV s^–1^, [K^+^] = 0.1 mol L^–1^, and pH = 1.0, produced the highest C_s_ value of 15.14 mF cm^–2^. Consequently, these conditions were selected for further testing. The Pareto chart in Figure 1b highlights the statistical significance of individual factors and their interactions concerning the response variable. Additionally, eq 1 demonstrates how these effects influence the C_s_. Positive effects indicate a favorable contribution to C_s_, while negative effects result in a decrease. The model achieved a strong fit with an R^2^ value of 0.998, confirming good data alignment at a 95% confidence level. The center-point triplicate exhibited a relative standard deviation of 16.7%.

**Figure 1:**
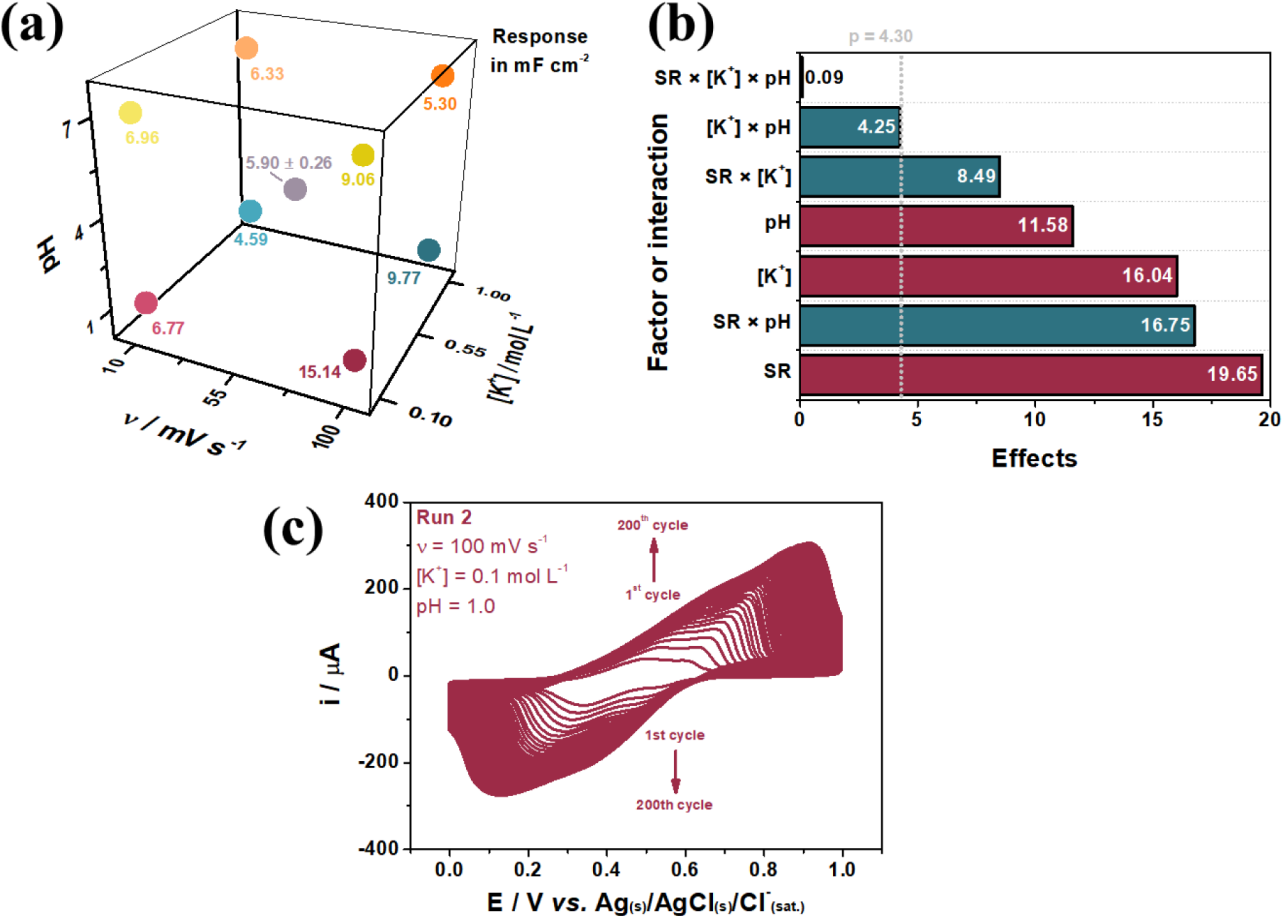
2^3^ factorial DOE geometric representation of the effects (a) and Pareto chart (b). Electrochemical conversion of the PLA/Gr/Ni to PLA/Gr/NiHCF based on the conditions of experiment 2 (c).

The Pareto chart (Figure 1b) reveals that the [K^+^] × pH interaction and the triple interaction (υ × [K^+^] × pH) have weights below the p-value threshold, indicating negligible contributions to the response. In contrast, the υ factor emerged as the most influential variable. As indicated by eq 1, higher υ values used in the preparation of the material correspond to increased C_s_. The next most significant interaction, υ × pH, and the individual [K^+^] factor show negative effects in eq 1, suggesting that higher C_s_ values are achieved at lower levels of these variables. The pH, while the fourth most statistically significant factor, exhibits a positive individual contribution to C_s_ according to eq 1. However, its synergistic interaction with υ, supported by lower υ and pH levels, dominates its effect.


1 $$C_{s}=5.924+0.108\upsilon -2.314\left[K^{+}\right]+0.119pH-0.039\upsilon \times \left[K^{+}\right]-0.012\upsilon \times pH+0.286\left[K^{+}\right]\times pH+0.0001\upsilon \times \left[K^{+}\right]\times pH-2.085PtCt$$

The υ × [K^+^] interaction, the least significant among the identified effects, negatively impacts C_s_ (as per eq 1). Despite υ being the most influential factor individually, its interaction with [K^+^] is antagonistic, meaning that higher C_s_ values depend on increasing υ and decreasing [K^+^]. The 2^3^ factorial DOE analysis supports the initial selection of conditions: υ = 100 mV s^–1^, [K^+^] = 0.1 mol L^–1^, and pH = 1.0, which correspond to Experiment 2. The CV response for the electrochemical conversion of the PLA/Gr/Ni precursor to PLA/Gr/NiHCF under these optimized conditions is shown in Figure 1c. This response displays a consistent increase in cathodic and anodic peak currents, confirming the nucleation and growth of NiHCF within the PLA/Gr matrix.

According to Vieira et al.,^33^ the υ significantly influences the size and distribution of the resulting particles, with higher υ favoring the nucleation and growth of smaller particles. This effect increases the surface area of NiHCF particles and provides more intercalation sites for ion diffusion. Similarly, a low pH during PBA formation is reported to enhance charge storage mechanisms. Zuo et al.^34^ demonstrated that acidic media promote the formation of K-rich PBA species, which offer greater ionic accommodation capacity. At pH = 1.0, the high mobility of H^+^ and the lower mobility of K^+^ create concentration gradients, these gradients facilitate K^+^ diffusion and its appropriate incorporation into the NiHCF framework.

### Electrochemical Behavior of the PLA/Gr/NiHCF Electrode

The CV profiles of the PLA/Gr and PLA/Gr/NiHCF electrodes at 10 mV s^–1^ in a 1.0 mol L^–1^ KNO_3_ solution are shown in Figure 2a. The inset highlights the box-shaped CV profiles of the PLA/Gr and PLA/Gr/Ni electrodes, which exhibit typical capacitive behavior without any redox-active species capable of accumulating and releasing charge through electrochemical reactions on the electrode surface. In contrast, the PLA/Gr/NiHCF electrode shows a significantly higher current response, characterized by two distinct redox peak pairs associated with the redox behavior of Fe^II^/Fe^III^ in two distinct NiHCF moieties, as widely reported in the literature.^20,22,34,35^

**Figure 2:**
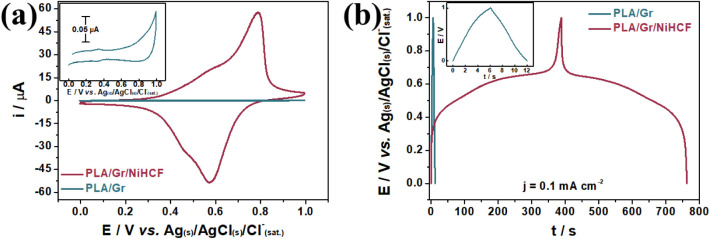
CV at 10 mV s^–1^ (a) and GCD at 0.1 mA cm^–2^ (b) of the PLA/Gr and PLA/Gr/NiHCF electrodes in a 1.0 mol L^–1^ KNO_3_ solution.

The first redox pair, observed at lower potentials, is attributed to electron transitions at iron sites accompanied by the intercalation of interstitial K^+^ ions in a K-free (or Ni-rich) fraction of the NiHCF, as described by eq 2. The second redox pair, located at higher potentials, is also linked to the redox activity of iron centers, but here, the K^+^ diffusion occurs in a K-rich NiHCF fraction, as represented by eq 3.^35^ Based on the areas under the CV profiles, the PLA/Gr/NiHCF electrode demonstrates a superior charge storage capability compared to PLA/Gr.


2a $$Ni_{1.5}\left[Fe^{III}{\left(CN\right)}_{6}\right]+K^{+}+e^{-}⇌KNi_{1.5}\left[Fe^{II}{\left(CN\right)}_{6}\right]$$

2b $$KNi\left[Fe^{III}{\left(CN\right)}_{6}\right]+K^{+}+e^{-}⇌K_{2}Ni\left[Fe^{II}{\left(CN\right)}_{6}\right]$$

Considering the negligible contribution of Gr to the total C_s_ relative to PLA/Gr/NiHCF, galvanostatic charge/discharge (GCD) measurements were conducted in a 1.0 mol L^–1^ KNO_3_ solution at a current density (j) of 0.1 mA cm^–2^, focusing solely on the predominant charge storage material (Figure 2b). The inset shows that the PLA/Gr electrode delivers stored charge within approximately 6 s. In contrast, the converted PLA/Gr/NiHCF electrode discharges over 375 s under the same conditions. The GCD profile of the NiHCF reveals two distinct electron transfer processes associated with the K-free and K-rich NiHCF fractions, occurring between 0.4 and 0.7 V. These processes are represented by potential plateaus over a broad range, indicative of mixed-controlled mechanisms. This behavior arises from a combination of diffusion and surface-adsorptive phenomena, characteristic of pseudocapacitive materials.^36^

To further analyze the current contributions from charge transfer phenomena, Dunn’s method was applied to the PLA/Gr/NiHCF electrode in a 1.0 mol L^–1^ KNO_3_ solution. This method evaluates the current response as a function of υ, distinguishing between capacitive contributions (proportional to υ) and diffusion-controlled contributions (proportional to the square root of υ).^37,38^ The material was analyzed by CV at υ of 1, 2, 5, 10, and 20 mV s^–1^ (Figure S3a). The resulting current was fitted using eq S2, where constants *k*_1_ and *k*_2_ represent the magnitudes of capacitive and diffusion-controlled contributions, respectively. Figure S3b shows the relative contributions of these mechanisms across all υ, confirming mixed charge storage behavior. The column graph highlights an increasing capacitive contribution with higher υ.

At the lowest υ (1 mV s^–1^), diffusion-controlled processes dominate due to sufficient time for K^+^ ions to diffuse into the inner interstitial sites of the electroactive material.^38,39^ The CV profile at this υ (Figure S3c) illustrates capacitive charge accumulation near the redox peaks represented by eq 2. Since the electrochemical reaction involves a redox transition accompanied by electron transfer, it exhibits surface-confined faradaic behavior, characteristic of pseudocapacitance. The K-free NiHCF moieties are likely more accessible due to the hydration and coordination of water molecules at defect sites in the PBA structure, which enhances K^+^ transport under an applied potential window. At a higher υ (20 mV s^–1^), the capacitive contribution becomes dominant, as shown in Figure S3d. This is particularly evident in the redox peak regions, further supporting the pseudocapacitive activity driven by surface-restricted faradaic processes. This observation underscores the efficient combination of diffusion and capacitive mechanisms in the charge storage behavior of the PLA/Gr/NiHCF electrode.

### Characterization

TGA was performed on materials scraped from PLA/Gr and PLA/Gr/Ni electrodes, with the results presented in Figure S4. The first noticeable weight loss for PLA/Gr/Ni occurred at an onset temperature (T_onset_) of 68 °C, corresponding to a 2.6% mass reduction. This event is attributed to the evaporation of coordinated water molecules from nickel acetate, which theoretically accounts for 3.6% of the material’s total weight. A major decomposition step, associated with oxidative degradation of the PLA matrix, was observed at T_onset_ values of 277 and 225 °C for PLA/Gr and PLA/Gr/Ni, respectively. The earlier onset in the PLA/Gr/Ni sample is likely due to the increased thermal conductivity from a higher graphite-to-PLA ratio, which accelerates heat transfer.^40,41^ Both materials exhibited similar overall mass losses: 62.7% for PLA/Gr and 61.1% for PLA/Gr/Ni. In PLA/Gr, an additional decomposition step at 428 °C accounted for a further 9.7% mass loss, likely related to more thermally stable PLA-derived species that had reacted with oxygen.^42^ This analysis indicates that the PLA content in PLA/Gr is approximately 70.8%. For PLA/Gr/Ni, a thermal plateau was observed starting at 394 °C and continuing up to about 550 °C, suggesting a PLA composition of 62.7%. This value is slightly higher than the theoretical estimate of 57.5%, possibly due to the concurrent formation of nickel oxide during the degradation process.^43^ A final weight loss observed around 600 °C in both materials marks the onset of graphite degradation. At 700 °C, the residual masses were 12.4% for PLA/Gr and 16.8% for PLA/Gr/Ni. The additional 4.4% residue in the latter may be attributed to nickel oxide, consistent with the 3% elemental nickel content in the composite filament.

The PLA/Gr/NiHCF electrode, along with its precursors (PLA/Gr/Ni and PLA/Gr), was analyzed by FTIR to confirm the presence of key components via characteristic vibrational modes. Figure 3a demonstrates the presence of PLA in all materials through distinct vibrational bands associated with various modes in its framework. The addition of other components did not result in significant shifts in these bands, indicating that PLA serves primarily as a physical support without significant chemical interactions. Table S3 provides the vibrational mode assignments and corresponding band positions of PLA.^44,45^ Notably, no bands associated with the vibrational modes of Gr were observed in the FTIR spectra due to the absence of a difference in the dipole moment of the graphitic bonds.

**Figure 3:**
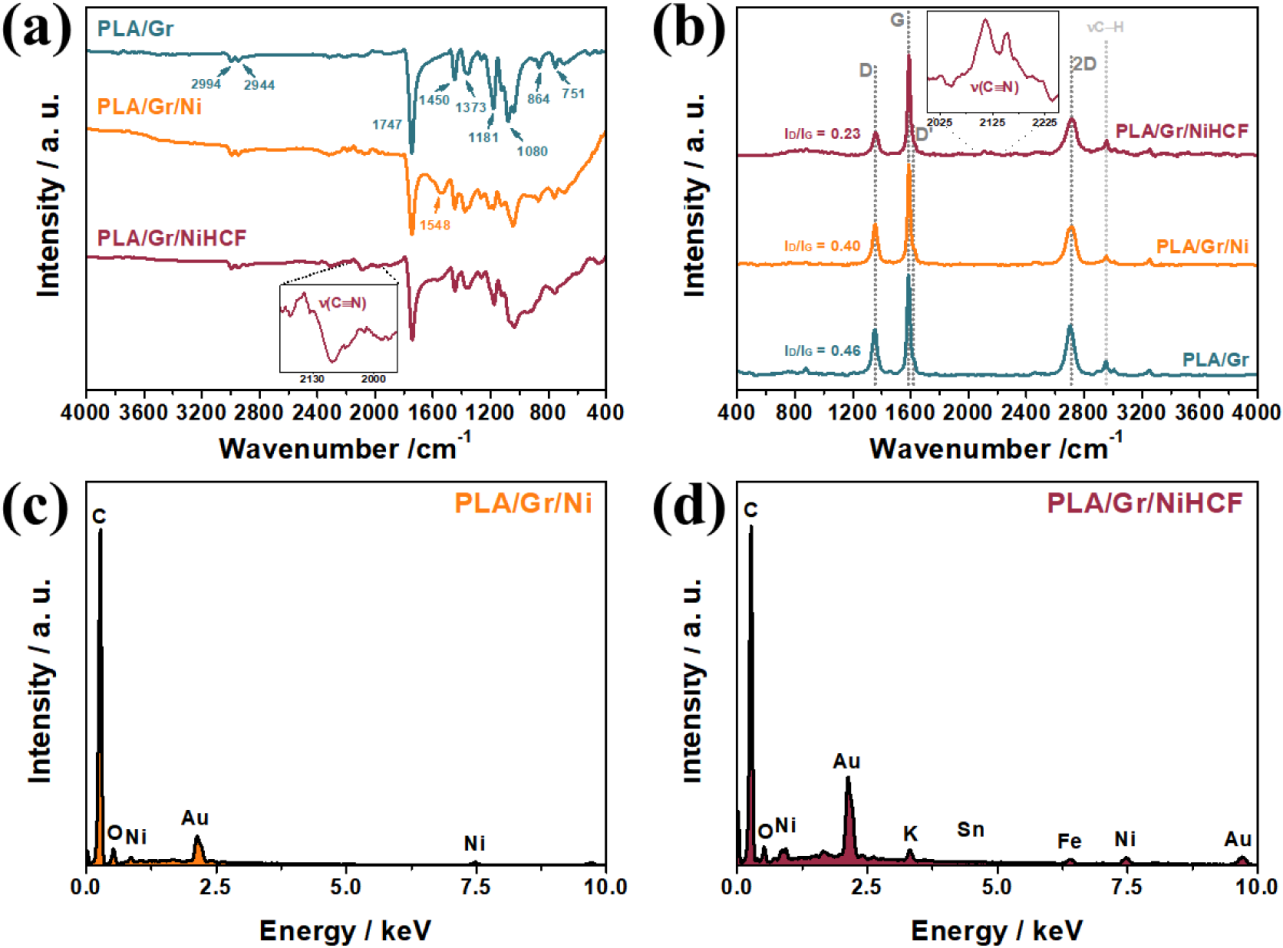
FTIR (a) and Raman (b) spectra of the PLA/Gr, PLA/Gr/Ni, and PLA/Gr/NiHCF materials. EDS spectra of the nickel-based electrodes PLA/Gr/Ni (c) and PLA/Gr/NiHCF (d).

The FTIR spectrum of the PLA/Gr/Ni electrode reveals a band at 1548 cm^–1^, attributed to the antisymmetric stretching of carboxylate anions from the nickel acetate precursor. This band persists in the PLA/Gr/NiHCF material, indicating residual acetate anions trapped within the insulating PLA matrix. Additionally, the formation of NiHCF is confirmed by the emergence of a band at 2089 cm^–1^, corresponding to the stretching vibrations of cyano group bridges in PBAs, specifically in Fe^II^–C≡N–Ni^II^ moieties when the metal species are in reduced forms.^46,47^

Raman spectroscopy was also performed on the PLA/Gr, PLA/Gr/Ni, and PLA/Gr/NiHCF electrodes using a 532 nm laser (Figure 3b). All spectra exhibit a band around 2954 cm^–1^, corresponding to the stretching of C–H bonds in PLA.^48^ The D, G, and 2D bands, located at approximately 1350, 1590, and 2700 cm^–1^, respectively, are characteristic of graphitic materials. The D band indicates structural defects in the sp^2^-carbon lattice, the G band represents the vibration of the sp^2^-carbon skeletal framework, and the 2D band reflects the stacking of graphene monolayers.^24,49,50^

No significant shifts in the graphitic bands were observed following the addition of nickel acetate or its conversion to PBA, highlighting the chemical robustness and inertness of graphite. The I_2D_/I_G_ ratios for all electrode materials were below 0.5, consistent with multilayered graphitic structures.^50^ However, the addition of nickel acetate decreased the I_2D_/I_G_ ratio from 0.46 to 0.30, and its conversion to PBA further reduced it to 0.24, suggesting that nickel acetate and hexacyanoferrate promote increased stacking of graphitic layers within the matrix.

The I_D_/I_G_ ratio, a measure of defect density in graphitic structures, also showed notable differences. The addition of nickel acetate slightly reduced the I_D_/I_G_ ratio from 0.46 to 0.40, implying a potential healing effect of the salt on defects in the carbonaceous material. Following electrochemical conversion to PLA/Gr/NiHCF, the I_D_/I_G_ ratio decreased significantly to 0.23, likely due to the deposition of NiHCF particles that fill defective sites, restoring the graphitic planes. Additionally, two new bands at 2111 and 2153 cm^–1^ were observed in the Raman spectrum of PLA/Gr/NiHCF, corresponding to cyano vibrational modes in Fe^II^–C≡N–Ni^II^ moieties,^47^ consistent with the FTIR findings.

The EDS spectra of the metal-containing materials (Figure 3c,d) confirmed the elemental composition, showing peaks for carbon (from PLA and Gr), nickel (in PLA/Gr/Ni), and both nickel and iron in PLA/Gr/NiHCF. These results validate the successful conversion of the precursor electrode into PLA/Gr/NiHCF and the retention of its structural integrity. Morphological characterization was performed via SEM, with images shown in Figure 4. The PLA and Gr components are evident in Figure 4a, where PLA appears as an amorphous, mushy material, while Gr exhibits rigid fragments with smooth surfaces and compacted plaques. The addition of nickel acetate (Figure 4b) introduced distinct contrasting spots, corresponding to dispersed clusters of nickel acetate salt of varying sizes trapped in the PLA/Gr matrix. Following electrochemical conversion, the PLA/Gr/NiHCF material (Figure 4c) displayed markedly different morphology, with uniformly distributed globular NiHCF nanoparticles across the surface. This reorganization enhances the material’s charge retention capabilities by facilitating physical and electrical interactions with the electrolyte solution.

**Figure 4:**
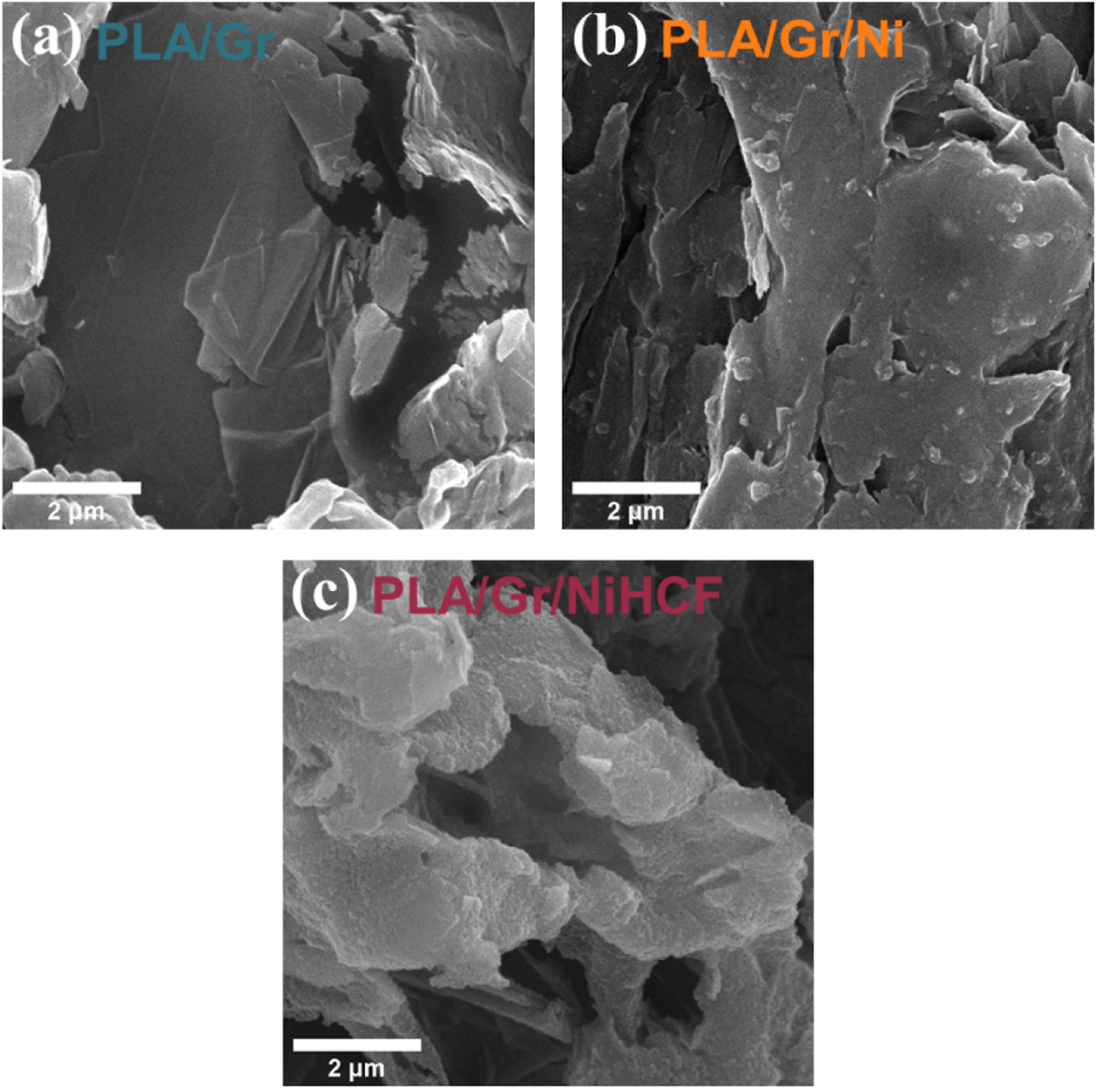
SEM images of the PLA/Gr (a), PLA/Gr/Ni (b), and PLA/Gr/NiHCF (c) materials.

EDS mapping of PLA/Gr/Ni and PLA/Gr/NiHCF electrodes surfaces was acquired from SEM images, and they are illustrated in Figure S5a,b, respectively. It was observed that, beyond the uniform distribution of the 10 nm-Au film coverage, all the other elements present spatial homogeneity. PLA/Gr matrices are constituted mostly of carbon, and PLA/Gr/Ni EDS mapping demonstrates well-dispersed Ni spots on the electrode’s surface. The same behavior is observed for the surface of the PLA/Gr/NiHCF electrode. Additionally, it is noted that the Fe spots distribution pattern follows the one from Ni, indicating successful and uniform conversion of the nickel sites to PBA structures. The converted electrode presented a Ni:Fe ratio of 2.26, suggesting the conversion process could induce the formation of a defective PBA structure composed of a large amount of [Fe­(CN)_6_]^3‑/4–^ vacancies. Furthermore, EDS measurements resulted in Ni/C ratios of 0.025 and 0.029 for PLA/Gr/Ni and PLA/Gr/NiHCF. Considering the 23.7% of Ni amount in nickel acetate tetrahydrate, the 12.5% Ni-loaded electrodes should possess a 0.03 Ni/C ratio, which confirms the initial composition is almost fully conserved in the functional electrodes.

### Electrochemical Performance of the PLA/Gr/NiHCF Electrode

The C_s_ of the PLA/Gr and PLA/Gr/NiHCF electrodes were evaluated using GCD measurements at current densities of 0.1, 0.2, 0.5, 1.0, and 2.0 mA cm^–2^ in a 1.0 mol L^–1^ KNO_3_ solution, calculated via Eq. S3.^32^ The GCD profiles of both electrodes are shown in Figure 5a,b, respectively. The PLA/Gr electrode displayed nearly ideal capacitive behavior (Figure 5a), indicative of fast charge and discharge processes. However, its low C_s_ at all current densities is attributed to purely electrostatic interactions between the electrode and electrolyte. At 0.1 mA cm^–2^, the highest C_s_ achieved was 0.58 mF cm^–2^, with a discharge time of 12 s.

**Figure 5:**
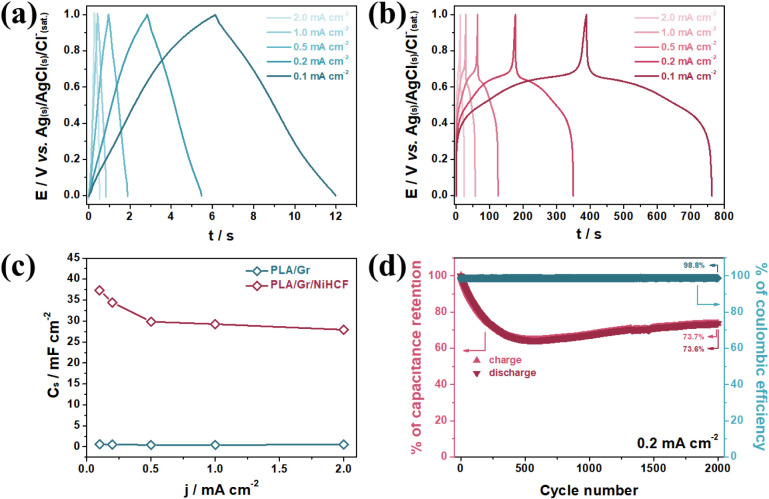
GCD profiles of the PLA/Gr (a) and PLA/Gr/NiHCF (b) in a 1.0 mol L^–1^ KNO_3_ solution under 0.1, 0.2, 0.5, 1.0, and 2.0 mA cm^–2^ current density. (c) C_s_ calculated from (a) and (b). (d) Capacitance retention and Coulombic efficiency for 2000 GCD cycles in a 1.0 mol L^–1^ KNO_3_ solution under 0.2 mA cm^–2^ current density.

In contrast, the PLA/Gr/NiHCF electrode exhibited superior performance, with a C_s_ of 37.33 mF cm^–2^ at the same current density, corresponding to a discharge time of 374 s. This substantial enhancement arises from faradaic processes involving the reduction of Fe^III^ sites in the two distinct structural moieties of NiHCF, as described in eq 2,3. The C_s_ values for both electrodes at all tested current densities are summarized in Figure 5c. While PLA/Gr showed minor variations (0.58 to 0.43 mF cm^–2^), PLA/Gr/NiHCF demonstrated consistently higher C_s_ (37.33 to 27.93 mF cm^–2^), reflecting the significant contribution of the NiHCF component.

Capacitance retention was assessed over 2000 GCD cycles at 0.2 mA cm^–2^ in a 1.0 mol L^–1^ KNO_3_ solution. The retained relative C_s_ values for each cycle are depicted in Figure 5d (red curve, left *y*-axis). A drop to ∼60% of the initial C_s_ was observed within the first 500 cycles, followed by a gradual recovery up to ∼1800 cycles. From this point onward, the capacitance stabilized, reaching 73.7% in the final cycle. This behavior is attributed to the structural conditioning of the PBA material: during initial cycles, hydrated K^+^ ions diffuse into interstitial channels, reorganizing coordinated water molecules and rearranging the crystalline structure. After this reorganization, the PBA achieves a stable configuration.^51,52^ Coulombic efficiency (CE) was calculated using eq S4 ^32^ and is presented in Figure 5d (blue curve, right *y*-axis). The PLA/Gr/NiHCF electrode demonstrated excellent charge/discharge reversibility, with an average CE of 98.7% over 2000 cycles.

EIS measurements for the PLA/Gr and PLA/Gr/NiHCF electrodes were conducted in a 1.0 mol L^–1^ KNO_3_ solution under open circuit potential (OCP) with a 10 mV potential amplitude over a frequency range of 100 kHz to 10 mHz. The resulting Nyquist diagrams are shown in Figure 6a, with equivalent circuits fitted in Figure 6b. Both electrodes exhibited semicircular responses, indicative of charge transfer processes at the electrode/electrolyte interface. The high-frequency *x*-axis extrapolation estimates the solution resistance (R_s_), while the low-frequency region provides the charge transfer resistance (R_ct_).^53,54^ PLA/Gr/NiHCF demonstrated a significantly lower R_ct_ of 20.04 kΩ compared to 325.0 kΩ for PLA/Gr—a 16-fold reduction—highlighting the enhanced charge transfer efficiency of the NiHCF-based electrode.

**Figure 6:**
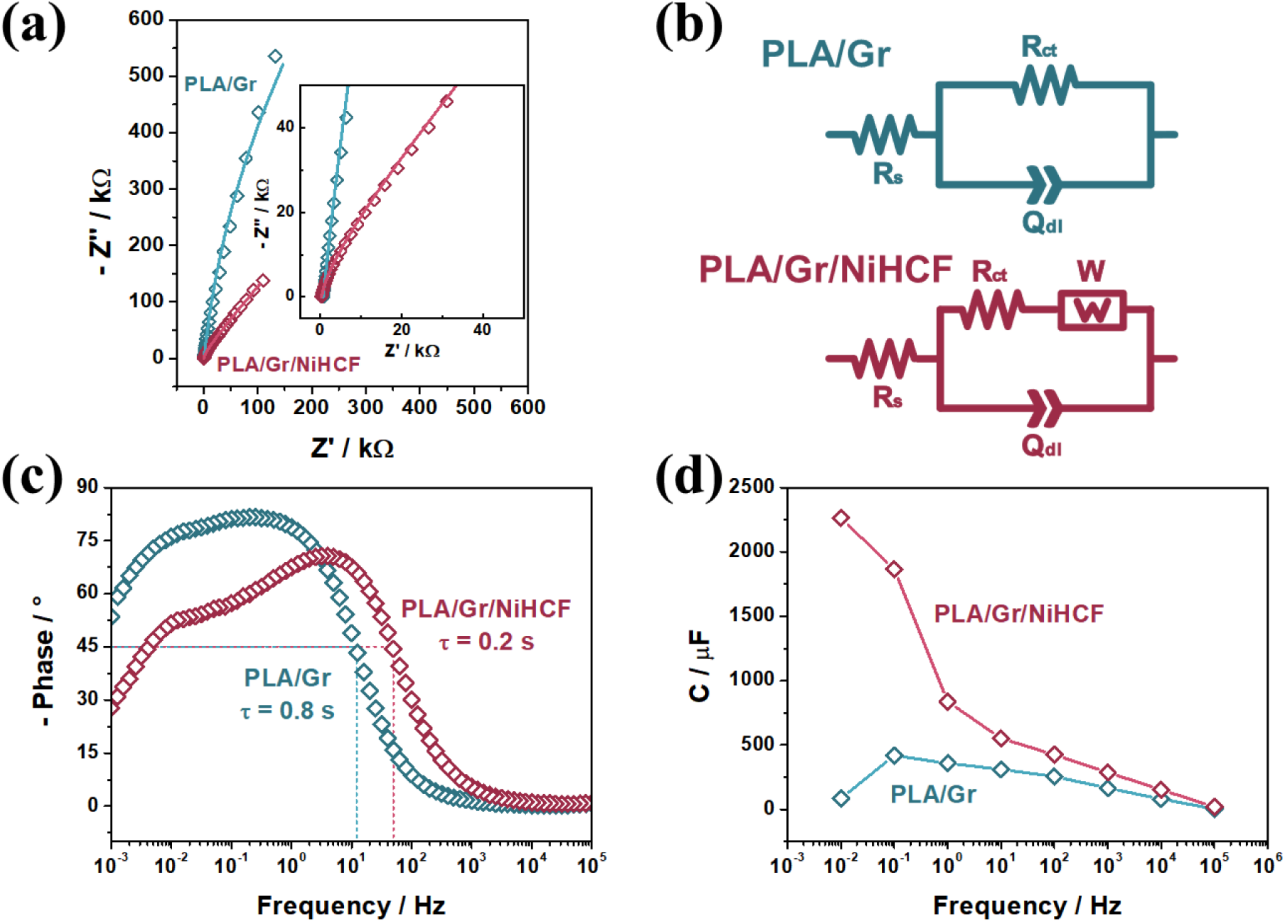
EIS Nyquist diagram (a) and fitted equivalent circuits (b). Bode spectra (c) and plot of capacitance versus frequency (d) of the materials. Conditions: OCP (10 mV amplitude) in a 1.0 mol L^–1^ KNO_3_.

The electrical double-layer capacitance (Q_dl_) was slightly lower for PLA/Gr/NiHCF (20.39 μΩ^–1^ s^N^) compared to PLA/Gr (23.60 μΩ^–1^ s^N^), reflecting minimal differences in double-layer formation. However, the N factor, which quantifies deviations from ideal capacitive behavior (with *N* = 1 indicating a perfectly smooth surface), was 0.89 for PLA/Gr/NiHCF and 0.93 for PLA/Gr. The lower N factor for PLA/Gr/NiHCF suggests that in situ NiHCF formation creates a rougher surface, which enhances electrode–electrolyte contact.

Bode spectra (Figure 6c) show phase angle versus frequency. The capacitor response frequency (*f*_0_), defined at a phase angle of −45°, marks the transition between capacitive and resistive behavior. The relaxation time (τ), calculated at *f*_0_ using eq S5 indicates the minimum time for 50% energy delivery efficiency. The PLA/Gr/NiHCF electrode exhibited a τ of 0.02 s, four times shorter than PLA/Gr (0.08 s), confirming the superior charge transfer efficiency of the PBA-based electrode.^55,56^ The capacitance versus frequency, calculated via eq S6 is shown in Figure 6d. PLA/Gr/NiHCF outperformed PLA/Gr, particularly at frequencies below 1 Hz, where the Warburg diffusion component (W) dominates. This reflects efficient charge transfer facilitated by K^+^ diffusion in the PBA structure, reinforcing the role of intercalation processes (eq 2,3) in charge storage mechanism.

### Electrochemical Performance of the Symmetrical System

A symmetrical two-electrode system was assembled using PLA/Gr/NiHCF electrodes as both cathode and anode in a 1.0 mol L^–1^ KNO_3_ solution. CV measurements at υ of 1, 2, 5, 10, and 20 mV s^–1^ were performed to assess the origin of the current contributions, calculated using the Dunn method (eq S2). The CV profiles are shown in Figure S6a. Unlike the three-electrode configuration, the symmetrical system exhibits a merged redox pair, combining the K-rich and K-poor species of the PBA. This redox pair corresponds to the faradaic process involving K^+^ intercalation. Figure S6b highlights the predominance of capacitive currents across all υ. The equivalence in composition, weight, and area of the cathode and anode in this configuration minimizes the diffusive current contribution from the bulk electrolyte, which is more prominent in three-electrode setups. At a low υ (1 mV s^–1^), Figure S6c shows partial capacitive contribution in the redox pair, while at a higher υ (20 mV s^–1^), Figure S6d demonstrates that the redox peaks are almost fully capacitive. These findings confirm the surface-confined redox activity of the material, indicating pseudocapacitive behavior of the PLA/Gr/NiHCF electrode in the two-electrode symmetrical system.

GCD measurements were conducted under different current densities in a 1.0 mol L^–1^ KNO_3_ solution, with the results presented in Figure 7a. The pseudocapacitive behavior observed in CV is also evident in the GCD profiles, which indicate mixed-controlled processes driven by the redox activity of both ″soluble″ and ″insoluble″ NiHCF moieties at the electrode surface. The C_s_ of the symmetrical PLA/Gr/NiHCF//PLA/Gr/NiHCF configuration were calculated using eq S7,^57^ with values of 40.4, 37.4, 32.5, 29.4, and 25.6 mF cm^–2^ obtained at current densities of 0.1, 0.2, 0.5, 1.0, and 2.0 mA cm^–2^, respectively (Figure 7b). The system’s specific energy (E_s_) and power (P_s_) densities were calculated via eq S8 and S9,^57^ and their correlation is depicted in the Ragone plot (Figure 7c). These results confirm the classification of the proposed symmetrical system as a supercapacitor device. Capacitance retention and CE were evaluated over 100 GCD cycles at a current density of 0.2 mA cm^–2^. Figure 7d shows a slight C_s_ retention drop of approximately 5% (left *y*-axis, red markers) and an average CE of 98.3% throughout the cycling process (right *y*-axis, blue markers). These results demonstrate the excellent charge–discharge reversibility and stability of the symmetrical system.

**Figure 7:**
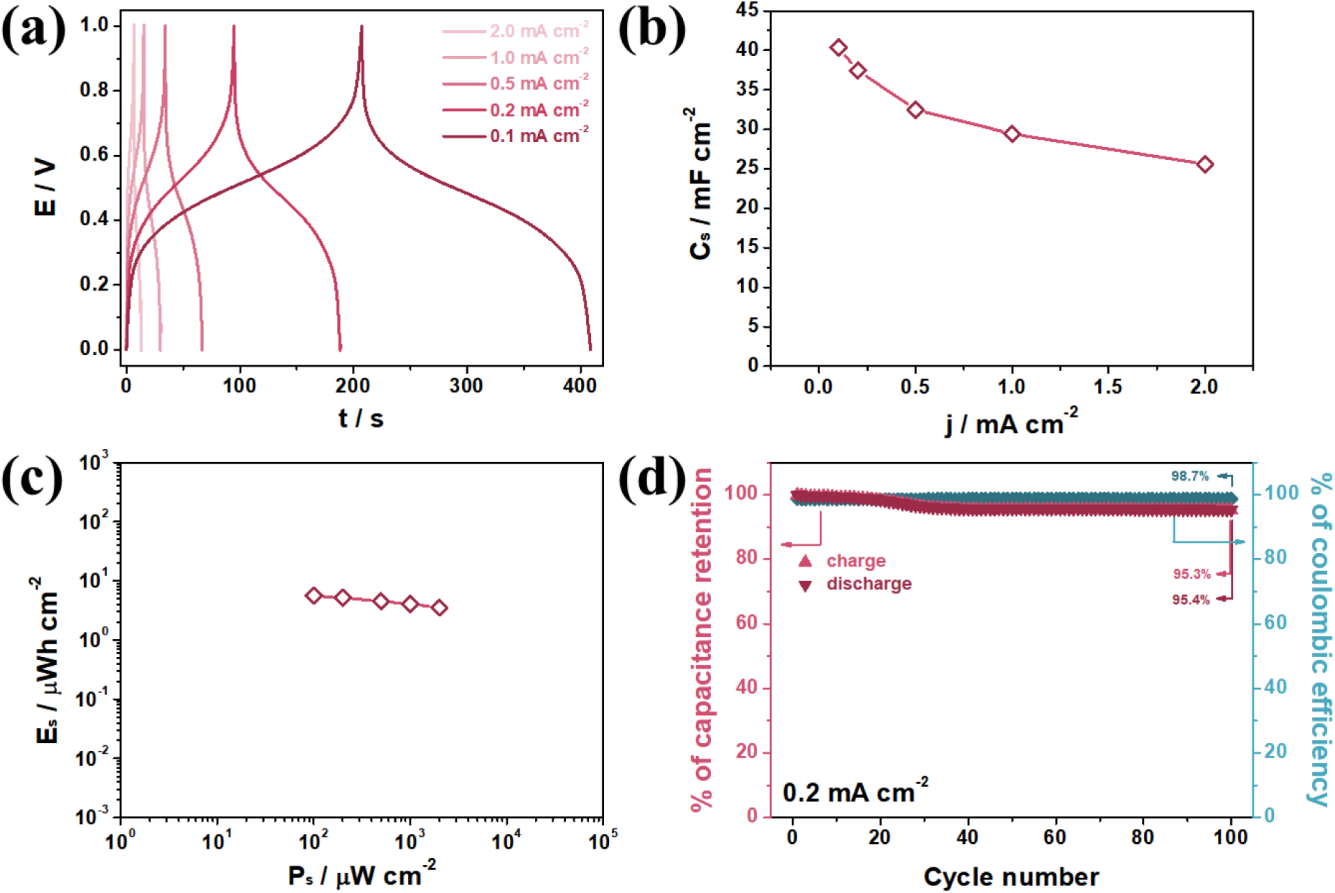
GCD profile of the PLA/Gr/NiHCF// PLA/Gr/NiHCF system in a 1.0 mol L^–1^ KNO_3_ solution under 0.1, 0.2, 0.5, 1.0, and 2.0 mA cm^–2^ current densities. (a) C_s_ response under the different current densities (b) Ragone plot of the symmetrical system (c). Capacitance retention and Coulombic efficiency for 100 GCD cycles in a 1.0 mol L^–1^ KNO_3_ solution under 0.2 mA cm^–2^ current density (d).

## Conclusions

This study demonstrates the successful development of a 3D-printed PLA/Gr/NiHCF electrode optimized using a factorial design approach. This methodology was key to systematically improving the electrode’s composition and performance while ensuring reproducibility. The simplicity of the fabrication process, particularly the in situ formation of NiHCF particles in the PLA/Gr matrix and the use of 3D printing, highlights the scalability and practicality of this approach. The electrode exhibited excellent pseudocapacitive behavior, high specific capacitance, and cycling stability in both three-electrode and symmetrical two-electrode systems. These results emphasize the potential of combining factorial design with accessible production techniques to create cost-effective, high-performance materials for sustainable energy storage applications.

## Supplementary Material



## Notes

ABS template printing parameters. Experiments, factors, and CV responses of the DOE. CV and current contributions obtained by Dunn’s Method for two-electrode and three-electrode systems. FTIR assignments for PLA vibrational modes. Summarized components results of the EIS electrical circuit fitting. General mathematical formulas (PDF)
