# Supplementary material for: Nickel-Loaded 3D-Printed Electrode for In Situ Electrochemical Conversion to a Prussian Blue Analogue: Synthetic Parameter Optimization for Pseudocapacitor Applications
Source: ACS Mater Au. 2025 May 28;5(4):675–86. doi: 10.1021/acsmaterialsau.5c00025 (PMC12257416; doi:10.1021/acsmaterialsau.5c00025)
Supplement: Supplementary file 1 [file mg5c00025_si_001.pdf]

Nickel-Loaded 3D-Printed Electrode for In Situ Electrochemical Conversion to a Prussian Blue Analogue: Synthetic Parameter Optimization for Pseudocapacitor Applications

Pedro H. S. Borges,[a] *Natália M. Caldas,*[b] *Lucas V. de Faria,*[b,c] *Rafael M. Dornellas,*[b] *Edson Nossol*[a,*]

[a] Institute of Chemistry, Universidade Federal de Uberlândia, Uberlândia 38408-902, Brazil

[b] Department of Analytical Chemistry, Institute of Chemistry, Universidade Federal Fluminense, Niterói 24020-141, Brazil

[c] Department of Analytical Chemistry, Institute of Chemistry, Federal University of Rio de Janeiro 21941-909, Brazil

* Email: enossol@ufu.br

Keywords: *pseudocapacitor, 3D printing, energy storage, nickel hexacyanoferrate, Prussian blue analogue*

Supporting Information

Figure S1. Three- **(a)** and symmetric two-electrode **(b)** systems photographic images.

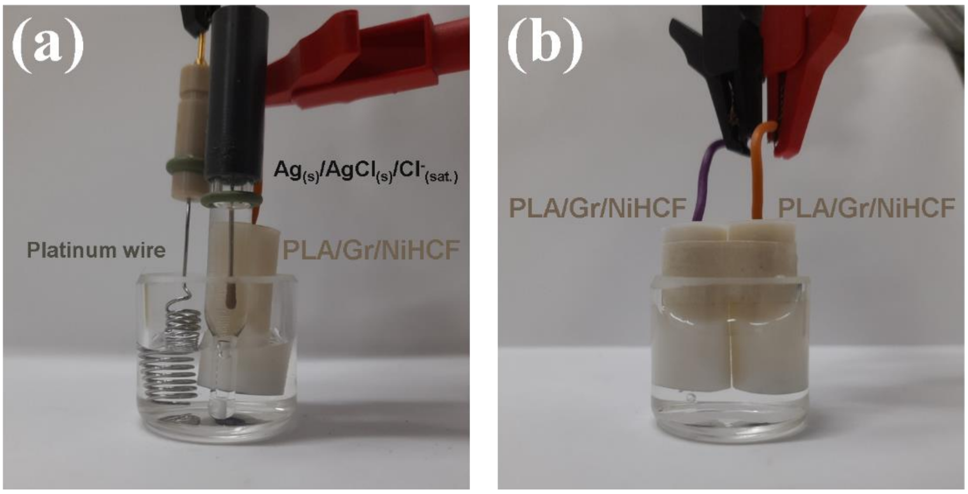

Table S1. Parameters employed in the ABS 3D-printed template

Printing parameter	Employed value
**Infill**	100%
**Number of perimeters**	3
**Printing speed**	100 mm s^{-1}
**Layer thickness**	0.3 mm
**Bed temperature**	110 °C
**Nozzle diameter**	0.4 mm
**Extrusion temperature**	230 °C

Table S2. Applied factors valued at each 2^3 DOE for PLA/Gr/NiHCF conversion optimization.

Experiment	Factor		
	υ / mV s^{-1}	[K$^+$] / mol L^{-1}	pH
**1**	10	0.1	1.0
**2**	100	0.1	1.0
**3**	10	1.0	1.0
**4**	100	1.0	1.0
**5**	10	0.1	7.0
**6**	100	0.1	7.0
**7**	10	1.0	7.0
**8**	100	1.0	7.0
**9**	55	0.55	4.0
**10**	55	0.55	4.0
**11**	55	0.55	4.0

$$C_s = \frac{\int j \ dV}{\upsilon \ \Delta V} \qquad \text{Equation S1}$$

Figure S2. CV profiles at 10 mV s^{-1} obtained for each 2^3 DOE experiment for PLA/Gr/NiHCF conversion optimization in a 1.0 mol L^{-1} KNO_3 solution.

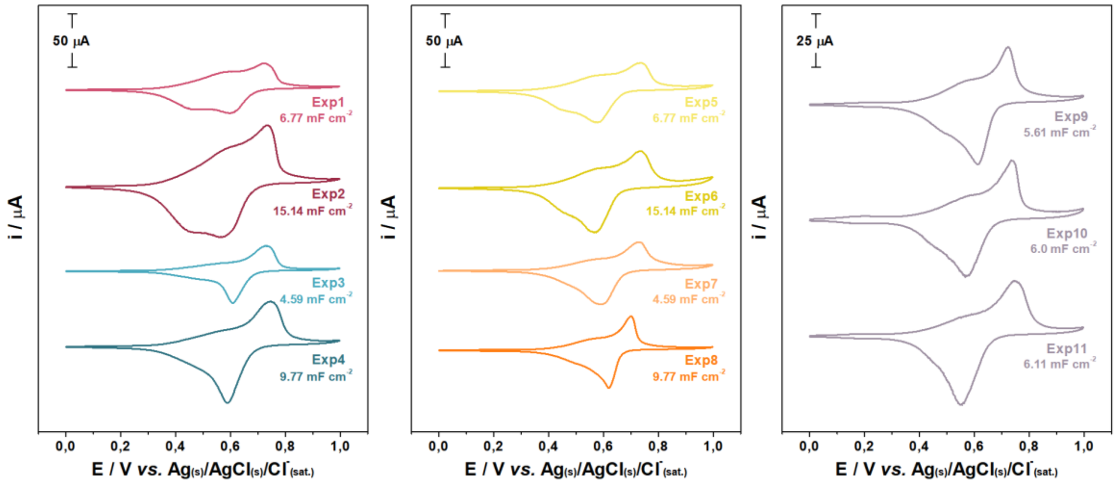

$$i = k_1 v + k_2 v^{1/2}$$ Equation S2

Figure S3. CV profiles of the PLA/Gr/NiHCF electrode in a 1.0 mol L^{-1} KNO_3 solution at 1, 2, 5, 10, and 20 mV s^{-1} **(a)**. Diffusion and capacitive contributions at each scan rate **(b)**. Charge origin distribution obtained by the Dunn method at 1 mV s^{-1} **(c)** and 20 mV s^{-1} **(d)**.

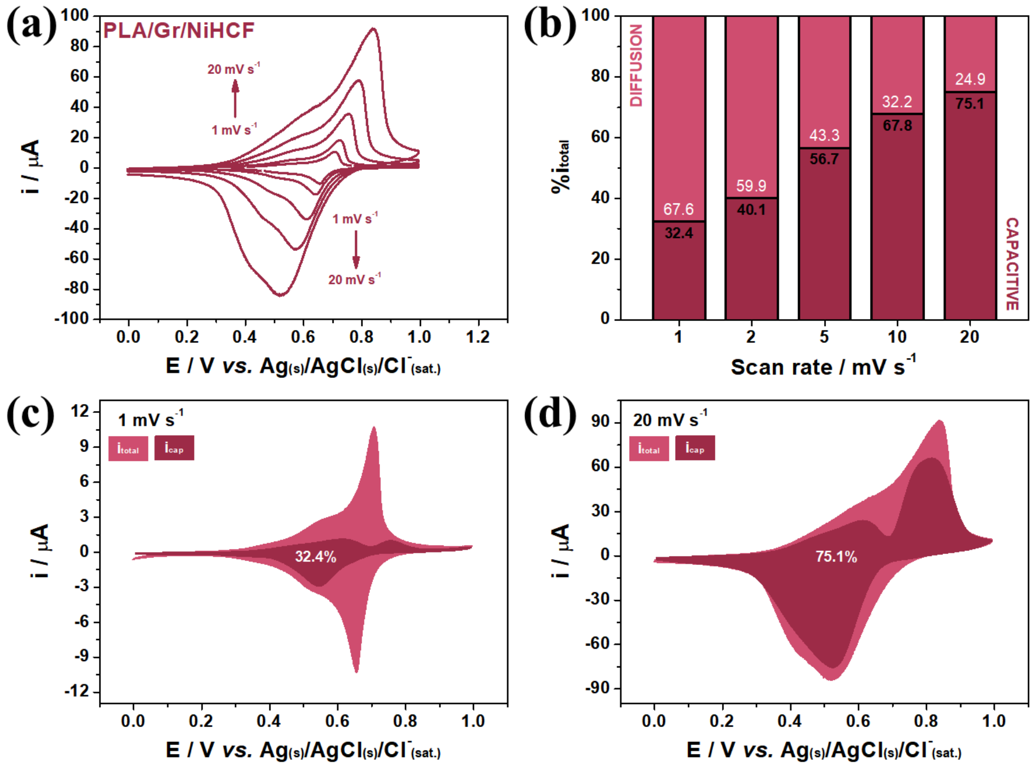

Figure S4. TGA of PLA/Gr control and PLA/Gr/Ni precursor electrodes.

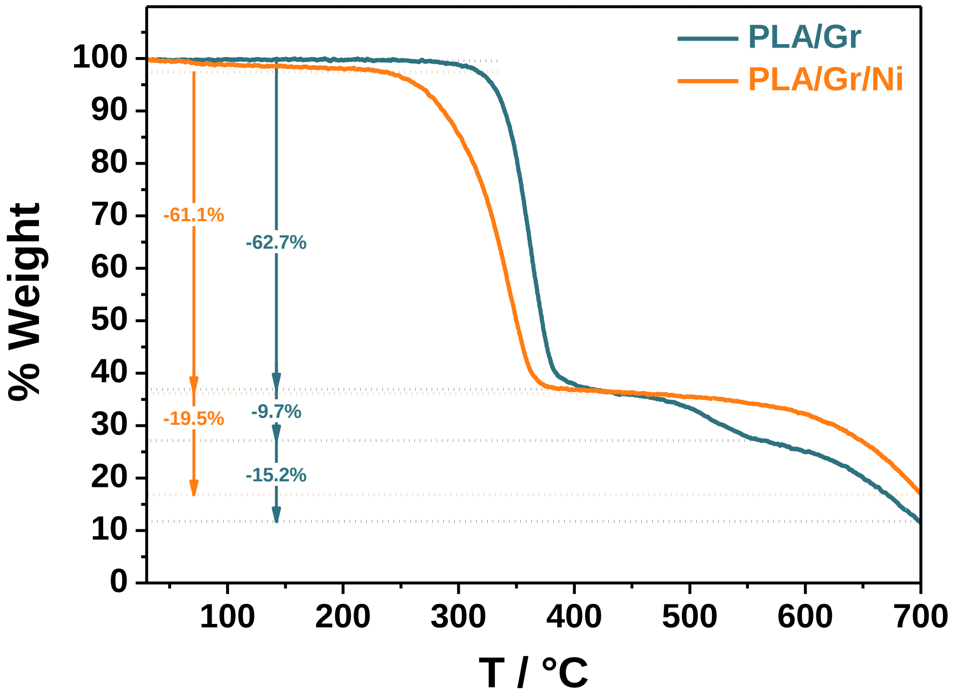

Table S3. FTIR bands assignments of the PLA.

Wavenumber / cm^{-1}	Assignment
752	δ(C=O)
863	υ(C–COO)
1035	υ(C–O–C)
1076	υ(C–O–C)
1125	$\upsilon(C–CH_3)$
1180	υ(C–O–C)
1263	υ(C=O)
1373	δ_s(CH)
1449	$\delta_{as}(CH_3)$
1746	υ(C=O)
2942	$\upsilon_s(C–CH_3)$
2990	$\upsilon_{as}(C–CH_3)$

Figure S5. Elemental EDS mapping of PLA/Gr/Ni **(a)** and PLA/Gr/NiHCF **(b)** electrodes.

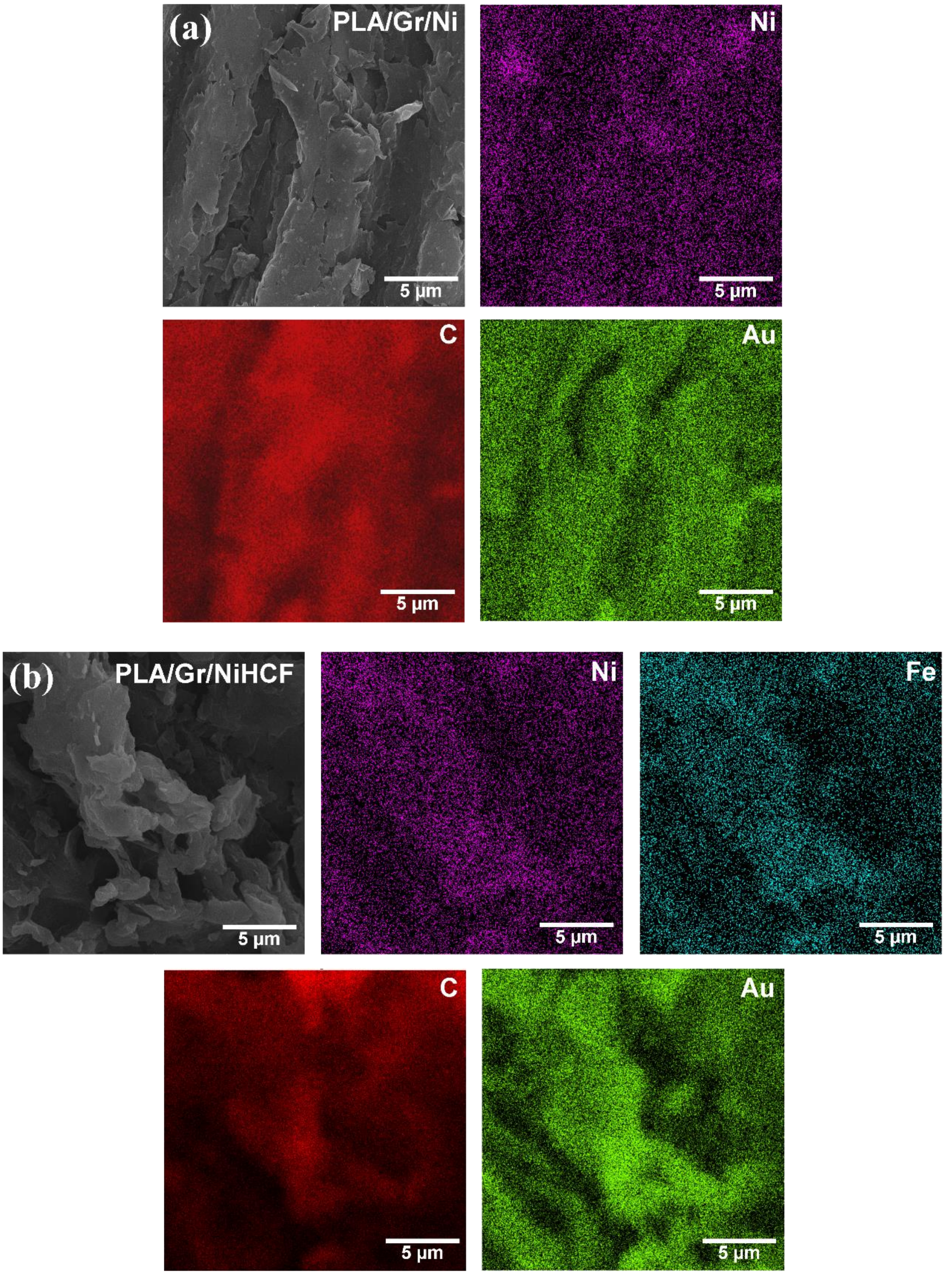

$$C_s = \frac{j\Delta t}{\Delta V}$$ Equation S3

$$CE = \frac{t_{discharge}}{t_{charge}} \times 100\%$$ Equation S4

Table S4. Equivalent circuit components parameters obtained from the EIS fitting.

**Material**	**R_s / Ω**	**R_{ct} / kΩ**	**Q_{dl} (N) / μ$Ω^{-1}$ s^N**	**W / μ$Ω^{-1}$ $s^{1/2}$**
**PLA/Gr**	639.66.	325.0	23.60 (0.93)	-
**PLA/Gr/NiHCF**	230.88	20.04	20.39 (0.89)	19.39

$$\tau = \frac{1}{f_0}$$ Equation S5

$$C = \frac{1}{2\pi f Z''}$$ Equation S6

Figure S6. CV profiles of the symmetrical system in a 1.0 mol L^{-1} KNO_3 solution at 1, 2, 5, 10, and 20 mV s^{-1} **(a)**. Diffusion and capacitive contributions at each scan rate **(b)**. Charge origin distribution obtained by the Dunn method at 1 mV s^{-1} **(c)** and 20 mV s^{-1} **(d)**.

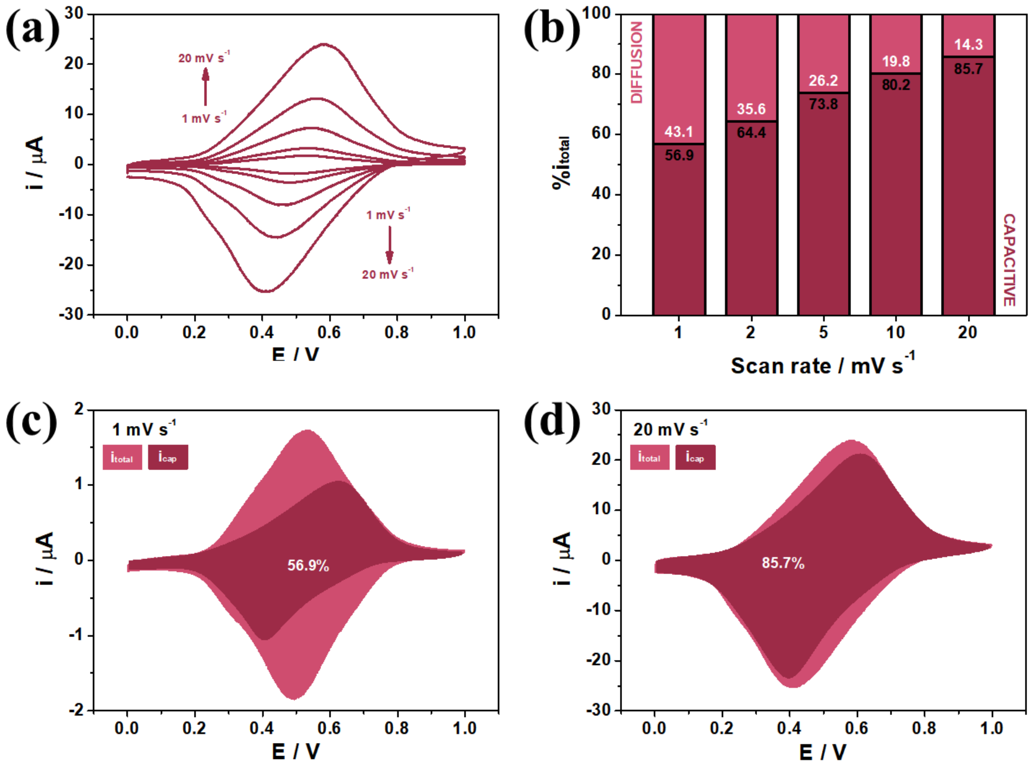

$$C_s = 2 \times \frac{j\Delta t}{\Delta V} \qquad \text{Equation S7}$$

$$E_s = \frac{C_s\,(\Delta V)^2}{2 \times 3600} \qquad \text{Equation S8}$$

$$P_s = \frac{E_s}{\Delta t} \times 3600 \qquad \text{Equation S9}$$
